# Supplementary material for: Osteopathic Manipulation of the Sphenopalatine Ganglia Versus Sham Manipulation, in Obstructive Sleep Apnoea Syndrom: A Randomised Controlled Trial
Source: J Clin Med. 2021 Dec 24;11(1):99. doi: 10.3390/jcm11010099 (PMC8745154; doi:10.3390/jcm11010099)
Supplement: Supplementary file 1 [file jcm-11-00099-s001.zip › jcm-1490242-supplementary.pdf]

Supplemental material:
Healthy Subjects Results

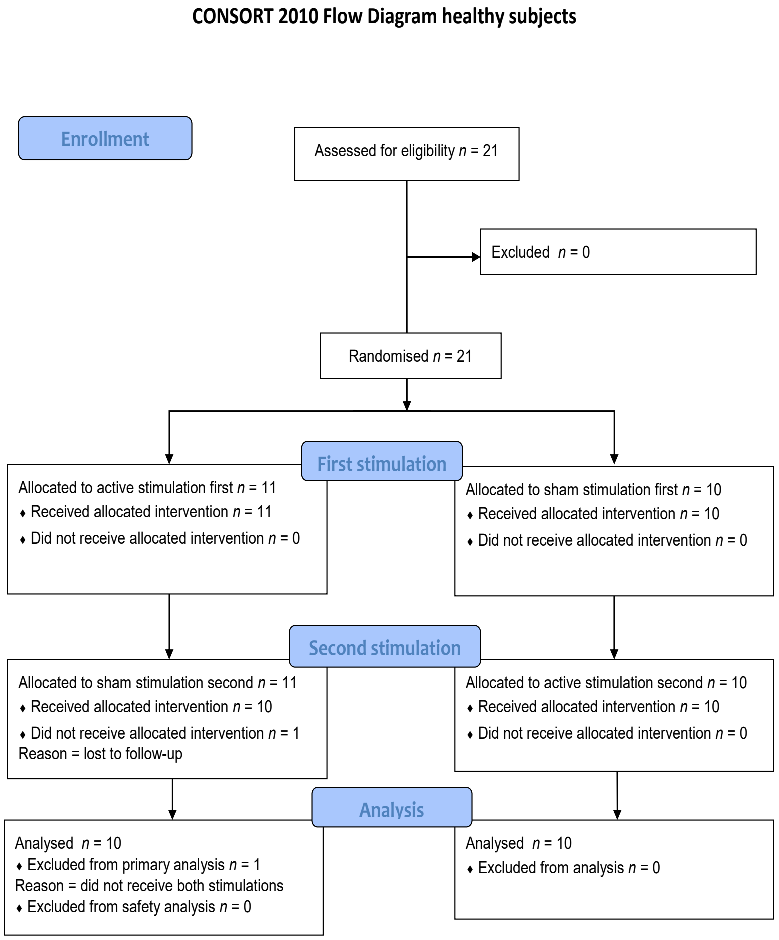

Figure S1. Consort Flow diagram for healthy subjects.

Table S1. Baseline data of healthy subjects before active manual stimulation of the sphenopalatine ganglion and before sham manual stimulation.

	Healthy subjects (n=21)	
**Age (years)**	25 [22; 31]	
Gender (Male/Female; n)	11/10	
	Before AM	Before SM
Weight (kg)	67 [59; 76]	67 [60; 76]
BMI (kg/m2)	22·3 [21·0; 26·9]	22·7 [20·9; 26·8]
ESS (/24)	7 [5; 10]	7 [6; 10]
PNIF (L/min)	125 [88; 155]	113 [68; 140]
Awake Pcrit*	-13·5 [-31·0; -8·1]	-17·6 [-21·4; -9·7]
